# Sertoli Cell Tumor (SCT) in a Captive Black Bear (*Ursus americanus*)

**DOI:** 10.3390/vetsci6040077

**Published:** 2019-09-26

**Authors:** Ahmed K. Elfadl, SunYoung Park, H M Arif Ullah, Soong-Hee Youn, Myung-Jin Chung, Ji-Yoon Son, Jae-Yeong Lee, Seoung-Woo Lee, A-Rang Lee, Su-Min Baek, Sul-Gi Jeon, Eun-Joo Lee, Il-Hwa Hong, Jin-Kyu Park, Kyu-Shik Jeong

**Affiliations:** 1 Department of Pathology, College of Veterinary Medicine, Kyungpook National University, Daegu 41566, Korea; ahmedpath@hotmail.com (A.K.E.); sunnypark78@gmail.com (S.P.); arifpha@ymail.com (H.M.A.U.); jin6850@naver.com (M.-J.C.); jiyoon1095@naver.com (J.-Y.S.); dud7776@gmail.com (J.-Y.L.); pyrk2000@gmail.com (S.-W.L.); leear3736@naver.com (A.-R.L.); suminbaek@gmail.com (S.-M.B.); jeonst77@naver.com (S.-G.J.); miffy525@hanmail.net (E.-J.L.); 2 Stem Cell Therapeutic Research Institute, Kyungpook National University, Daegu 41566, Korea; 3 Samsung Everland Zoological Garden, Yongin-si, Gyeonggido 17203, Korea; hapysh3@naver.com; 4 Department of Veterinary Pathology, College of Veterinary Medicine, Gyeongsang National University, Jinju 52828, Korea; ihhong@gnu.ac.kr

**Keywords:** Sertoli cells, testes, Ursidae, neoplasm, vimentin

## Abstract

A black bear of 29-year-old (*Ursus americanus*) died unexpectedly in captivity without any gross lesions or clinical signs. We identified a firm, lobulated, yellowish tan, and well-circumscribed mass embedded inside the testicular tissue at the time of necropsy. The tumor sections exhibited soft necrotic and hemorrhagic areas beneath its capsule. Histologically, the tumor comprised Sertoli cells arranged in tubules and solid sheets supported by prominent fibrous connective tissues. The Sertoli cells were positive for vimentin and ER-β expression, whereas it showed negative staining for inhibin-α, cytokeratin 19, and S-100. To the best of our knowledge, this is the rare case report of testicular Sertoli cell tumor in black bear.

## 1. Introduction

Based on literature reports, testicular tumors are most commonly observed in dogs. Seminomas are the most prevalent testicular neoplasms in elderly horses, whereas teratoma is more frequently diagnosed in young horses. Cats, boar, rams, and bucks are rarely affected by testicular tumors. However, both seminoma and Sertoli cell tumors have been observed in these species [1]. In the family Ursidae, the reported cases of neoplasms include extrahepatic biliary carcinoma in sloth bears, thyroid gland tumors, or squamous cell carcinomas of the larynx in a North American black bear, mandibular osteoma, papillary cyst adenocarcinoma of the mammary gland, or mucinous cholangiocarcinoma in a Himalayan brown bear [1,2,3,4,5,6].

## 2. Case Presentation

A testicle tissue sample from a black bear (*Ursus americanus*) at the Everland Zoo in Seoul, South Korea was sent to the veterinary pathology laboratory at Kyungpook National University for pathological examination. The bear was emaciated and died without any other abnormality at 29 years of age. 

**Figure 1:**
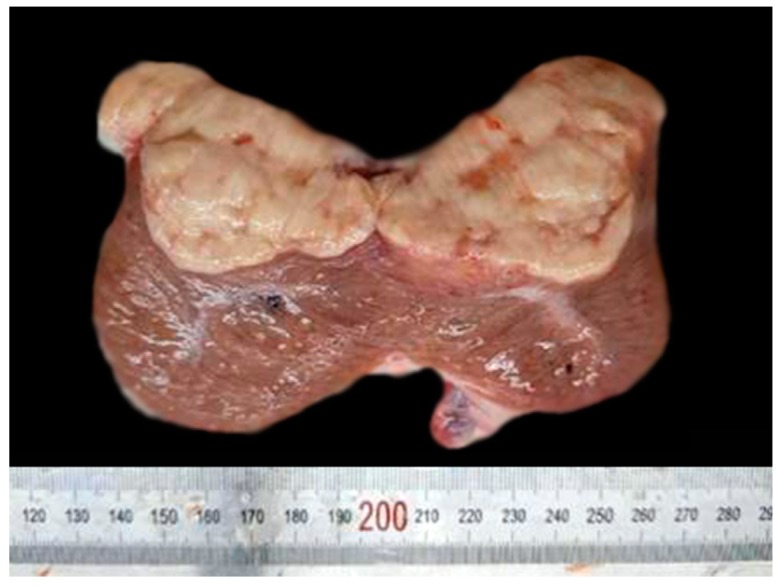
Cross section of the testicular tumor isolated from a black bear showing yellow, tan, and lobulated neoplasm, which is isolated from a nearby tissue by a fibrous capsule.

Gross description of the lesion was followed by routine processing, hematoxylin and eosin (H&E) staining of 5-μm thick sections. Markers including ER-α, ER-β, inhibin-α, cytokeratin 19, S-100, and vimentin immunoglobulins were used to determine the type of tumor. Gross examination of the tumor showed a firm, lobulated, yellow tan, and well-circumscribed mass embedded inside testicular tissue (Figure 1). Section of the tumor revealed that the neoplasm had soft necrotic areas with hemorrhage beneath the capsule.

**Figure 2:**
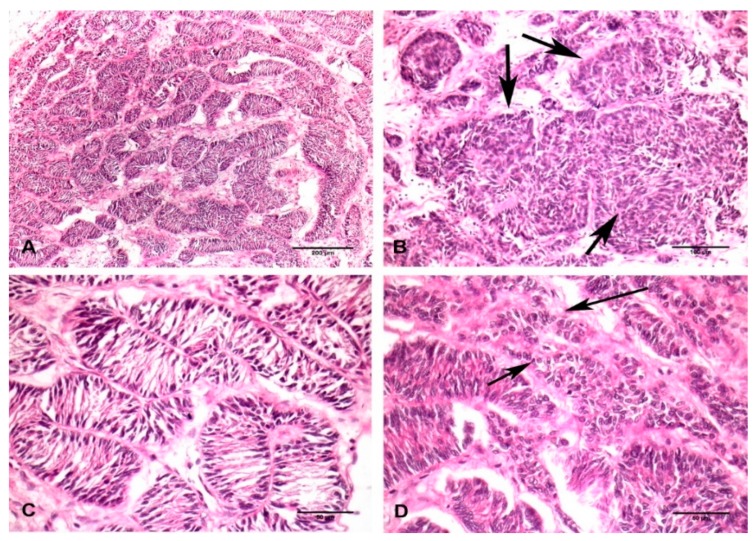
Histopathological picture of Sertoli cell tumor isolated from the testicle of a black bear. (**A**) General overview of Sertoli cells arranging in a tubular pattern with an occluded lumen. H&E staining. Bar = 200 µm. (**B**) Several tubules containing Sertoli cells merged together forming multiple large round masses of varied sizes (indicated by arrow). H&E staining. Bar = 100 µm. (**C**) Spindle-shaped Sertoli cells showing dark nuclei and small amounts of cytoplasm, these cells formed a bridge across the inner sides of tubules. H&E staining. Bar = 50 µm. (**D**) Well-differentiated Sertoli cells showing round-to-oval nuclei forming irregular shaped tubules (indicated by arrow). H&E staining. Bar = 50 µm.

Histologically, the tumor consisted of tubular and solid proliferation of Sertoli cells, supported by a fibrous connective tissue (Figure 2A). The tubules varied in size and generally lacked a lumen except in a limited area. In some areas, multiple tubules merged together to form large round nests of Sertoli cells, an interstitial space isolated these nests from the surrounding tissues (Figure 2B). Individual cells were spindle-shaped and tall, showing intensely stained, thin, spindle nuclei that lacked nucleoli. Interestingly, spindle-shaped Sertoli cells formed a bridge extending from one side of the inner tubules to the other side (Figure 2C). Occasionally, a few well-differentiated Sertoli cells were observed to have a large round nuclei and large amounts cytoplasm (Figure 2D). Hyalinized and dilated blood vessels were observed in tumor stroma. 

**Figure 3:**
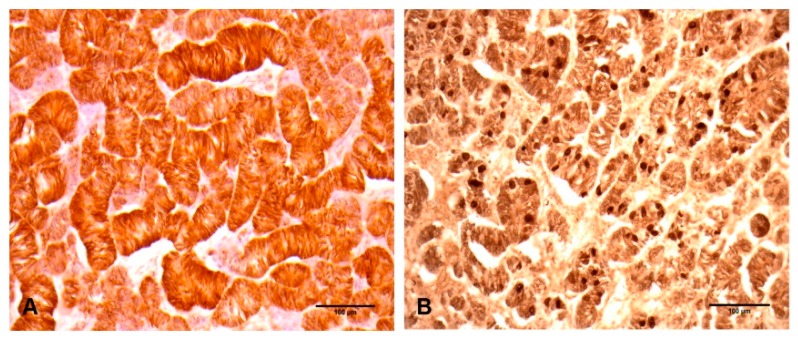
Immunohistochemical properties of Sertoli cell tumor isolated from the testicle of a black bear. (**A**) All tumor cells showed very strong positive reaction to vimentin immunoglobulins. Bar = 100 µm. (**B**) Numerous Sertoli cells showed a strong positive reaction to ER-β. Bar = 100 µm.

For immunohistochemistry, tissue sections were deparaffinized, rehydrated, blocked with 3% hydrogen peroxide in methanol, and transferred into a citrate buffer (pH 6.0) and microwave treated for antigen retrieval. Washing in phosphate-buffered saline (PBS), sections were incubated with the primary antibody for 1 h at room temperature. Then, antigen-antibody complex was visualized by an avidin-biotin peroxidase complex solution (ABC kit, Vector Laboratories, Bulingame, CA, USA) and followed using diaminobenzidine (DAB). Immunohistochemical analysis revealed that the neoplasm was positive for vimentin (Figure 3A) and ER-β immunoglobulins (Figure 3B), whereas cytokeratin 19, S-100, and inhibin-α were negative.

## 3. Discussion

Sex cord stromal tumors are a rare testicular tumor, accounting for 4% of all testicular neoplasms of human adult males. Sertoli cell tumors have a lower prevalence, representing 0.4–1.5% of testicular neoplasms [7]. Sertoli cell tumors are common in elder or cryptorchid dogs [8,9]. Generally, Sertoli cell tumors are benign. However, a few reports have described malignancy in dogs [8,10,11]. 

In the literature, only nine neoplasms have been reported in the Ursidae family, but none of them were observed in the testicles. Moreover, the bear was in captivity, subjected to disease monitoring and exhibited no signs on unexpected death at the age of 29. The testicular tumor was discovered during necropsy examination and was unlikely to be the cause of death. No one familiar with the animal observed any signs of feminization or alopecia, which are common clinical manifestations of Sertoli cell tumors. Lack of these signs could be owing to solitary captivity of the bear because feminization is most evident in the presence of another animal and is based on the interaction between the two animals. 

In dogs, the expression profile of Sertoli cell markers were predominantly consistent with testicular neoplasms [12,13,14]. Canine Sertoli cell tumors express vimentin but not cytokeratin, desmin, or inhibin-α [12,14]. 

In the present case, Sertoli cell showed a strong expression of vimentin in all parts of the neoplasm, whereas cytokeratin 19 and inhibin-α were negative. Interestingly, this expression pattern is consistent for Sertoli cell tumors in dogs. Sertoli cell within the bear’s neoplasm showed a strong positive expression for ER-β. This could be explained by the absence of inhibin-α expression, which regulates paracrine function of testicles by inhibiting follicle stimulating hormone and subsequently preventing estradiol expression [15].

## 4. Conclusions

To the author’s knowledge, this is an uncommon case of Sertoli cell tumors in an American black bear.
